# Supplementary material for: Transcriptome analyses reveal tau isoform-driven changes in transposable element and gene expression
Source: PLoS One. 2021 Sep 29;16(9):e0251611. doi: 10.1371/journal.pone.0251611 (PMC8480850; doi:10.1371/journal.pone.0251611)
Supplement: S1 Raw images — (PDF) [file pone.0251611.s001.pdf]

Cytoplasmic fraction | Nuclear fraction

3R tau primary antibody

50 kDa

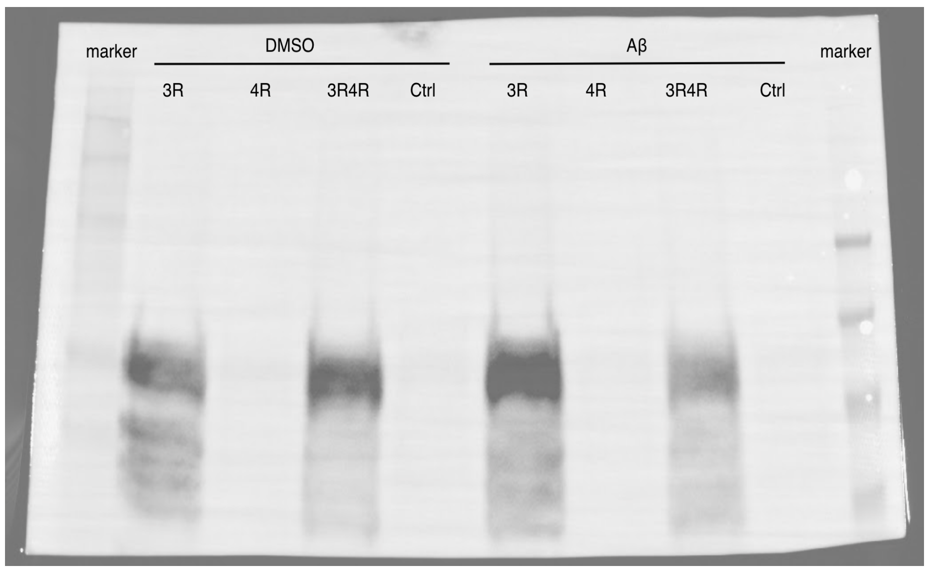

45 kDa

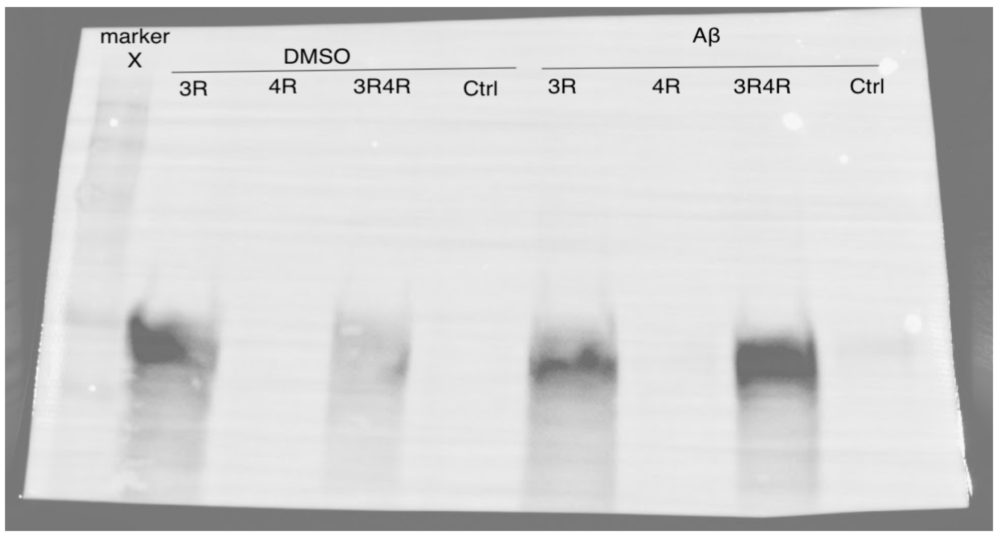

H3 primary antibody

20 kDa

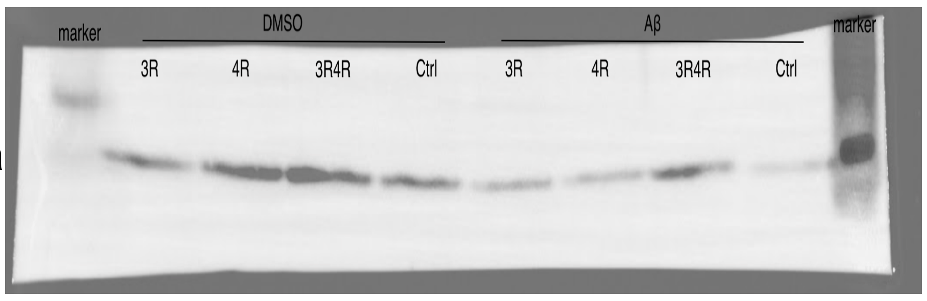

17 kDa

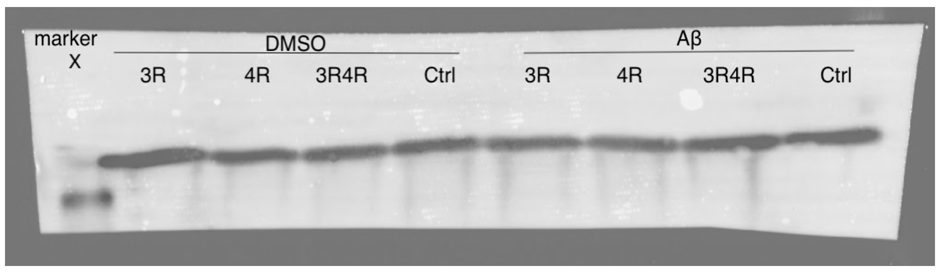

GAPDH primary antibody

33 kDa

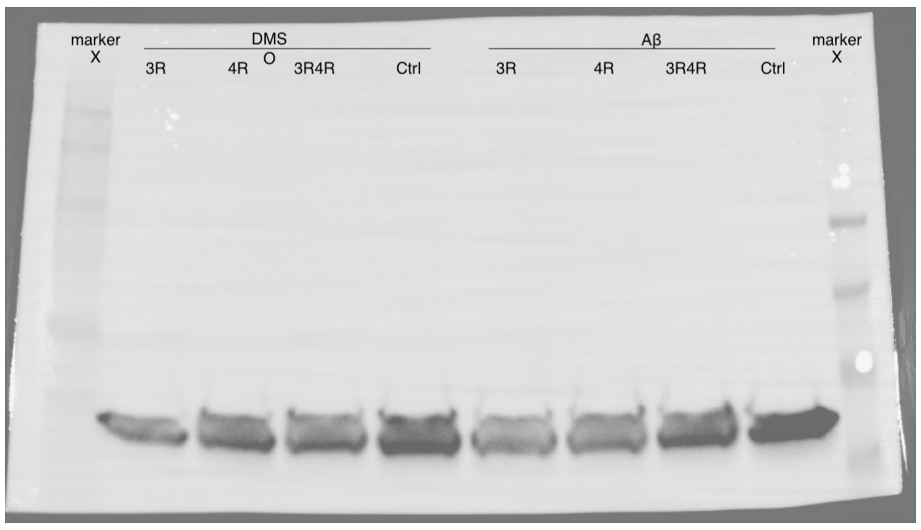

35 kDa

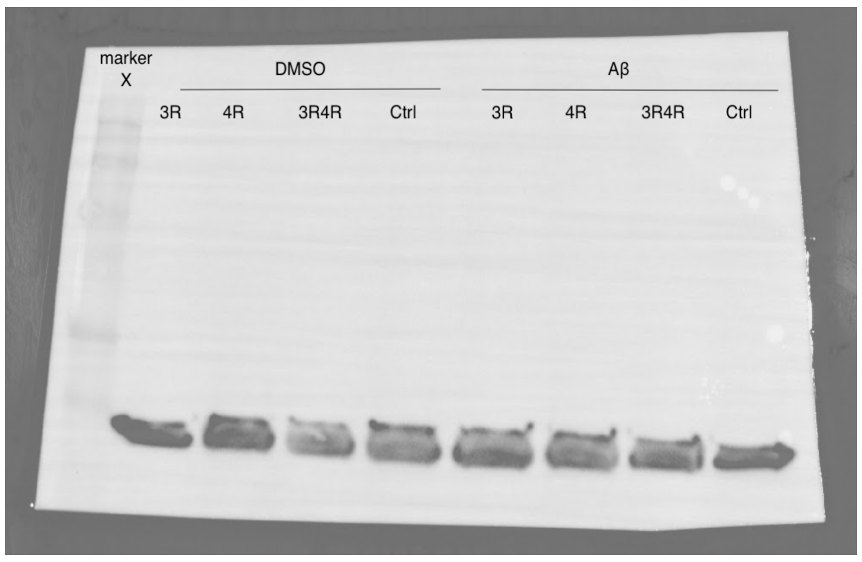

* All blots shown on this page were used in Fig 1A; top left and top right images.

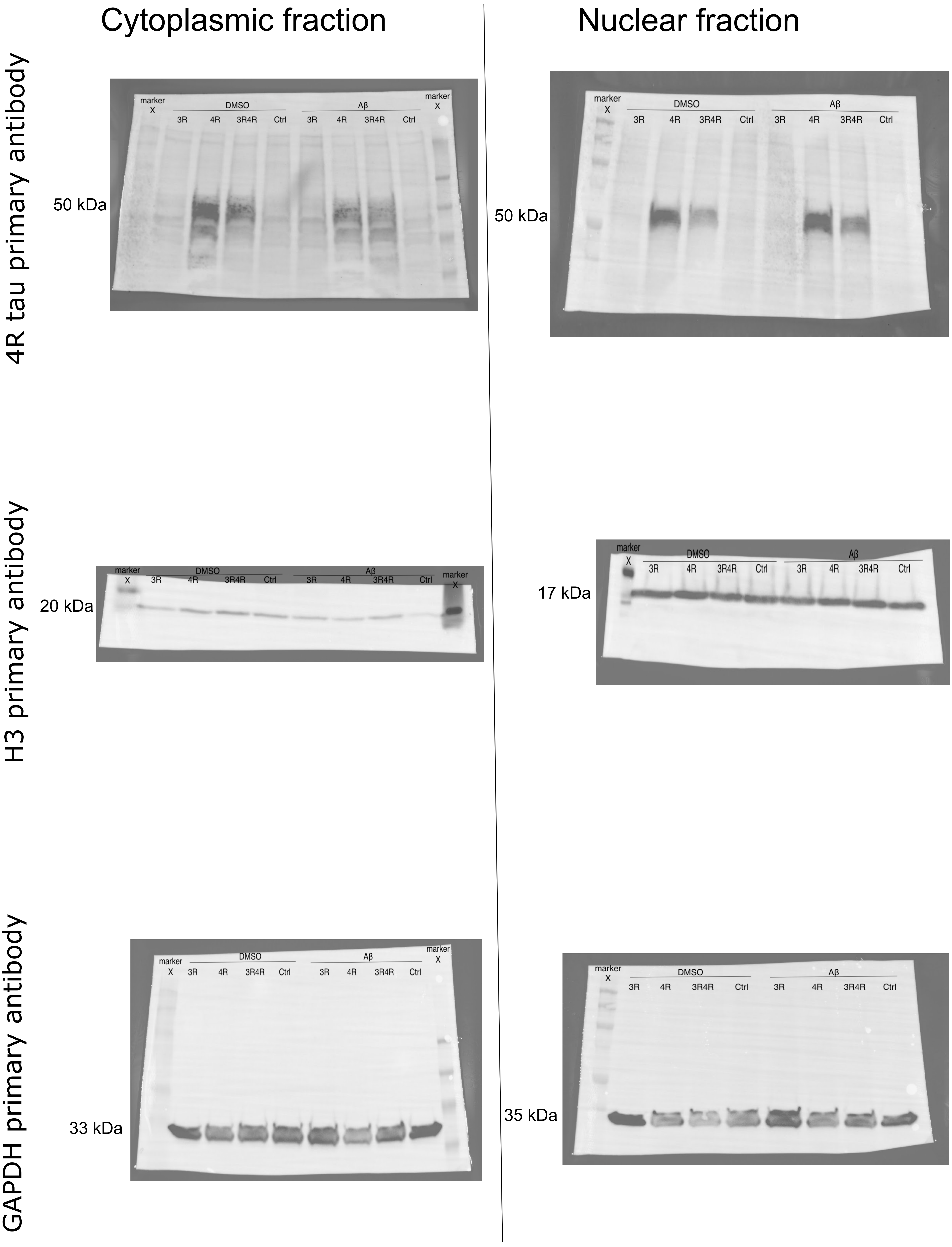

* All blots shown on this page were used in Fig 1A; bottom left and bottom right images.
