# Supplementary material for: Transcriptome analyses reveal tau isoform-driven changes in transposable element and gene expression
Source: PLoS One. 2021 Sep 29;16(9):e0251611. doi: 10.1371/journal.pone.0251611 (PMC8480850; doi:10.1371/journal.pone.0251611)
Supplement: S1 File — (ZIP) [file pone.0251611.s005.zip › S4_Code_and_Quality_Control_Data/examples_of_code_used.docx]

**Examples of code used to analyze RNA-seq data:**

BBduk:

~/DIRECTORY/bbduk/bbmap/bbduk.sh -Xmx1G \

in1=~/DIRECTORY/not_clean/${FILE}.r1.fastq.gz \

in2=~/DIRECTORY/not_clean/${FILE}.r2.fastq.gz \

out1=~/DIRECTORY/clean_${FILE}.r1.fastq.gz \

out2=~/DIRECTORY/clean_${FILE}.r1.fastq.gz \

ref=~/DIRECTORY/bbduk/bbmap/adapters.fa ktrim=r k=31 mink=11 hdist=1 tpe tbo

done

STAR:

*To create .bam files used for featureCounts input:*

STAR --genomeDir ~/DIRECTORY/genome_index \

--runThreadN 14 \

--readFilesCommand gunzip -c \

--readFilesIn ${FILE}.r1.fastq.gz ${FILE}.r2.fastq.gz \

--outSAMtype BAM SortedByCoordinate \

--outFileNamePrefix ~/DIRECTORY/BAMs/${FILE} \

--outSAMunmapped Within \

--outSAMattributes Standard

*To create .bam files used for TEcount and TElocal input:*

STAR --genomeDir ~/DIRECTORY/genome_index \

--runThreadN 14 \

--readFilesCommand gunzip -c \

--readFilesIn ${FILE}.r1.fastq.gz ${FILE}.r2.fastq.gz \

--outSAMtype BAM Unsorted \

--outFileNamePrefix ~/DIRECTORY/TE_${FILE} \

--outSAMunmapped Within \

--outFilterMultimapNmax 100 \

--winAnchorMultimapNmax 200 \

featureCounts:

*Human brain samples:*

featureCounts -p -B -t exon -s 0 -g gene_id -a Homo_sapiens.GRCh38.98.gtf -o TITLE.txt *.bam -T 12

*SH-SY5Y samples:*

TEcount:

*Human brain samples:*

TEcount -b ${FILE} --GTF Homo_sapiens.GRCh38.98.gtf --TE GRCh38_Ensembl_rmsk_TE.gtf --format BAM --stranded no --project ${FILE} --mode multi

*SH-SY5Y samples:*

TEcount -b ${FILE} --GTF Homo_sapiens.GRCh38.98.gtf --TE GRCh38_Ensembl_rmsk_TE.gtf --format BAM --stranded reverse --project ${FILE} --mode multi

TElocal:

*Human brain samples:*

~/TElocal-master/telocal -b $FILE --GTF Homo_sapiens.GRCh38.98.gtf --TE GRCh38_rmsk_TElocus.ind --stranded no --project TElocal_$FILE --mode multi -i 100

*SH-SY5Y samples:*

~/TElocal-master/telocal -b $FILE --GTF Homo_sapiens.GRCh38.98.gtf --TE GRCh38_rmsk_TElocus.ind --stranded reverse --project TElocal_$FILE --mode multi -i 100

DESeq2 linear models:

*Human brain samples:*

dds <- DESeqDataSetFromMatrix(sorted_counts, coldata, ~Gender + Source + FLOWCELL + Diagnosis)

*SH-SY5Y samples:*

dds <- DESeqDataSetFromMatrix(countData = counts,

colData = coldata,

design = ~conditions)

#where ‘conditions’ refers to the infection and treatment, e.g. “LV-3Rtau_with_DMSO”.
