# Supplementary material for: Transcriptome analyses reveal tau isoform-driven changes in transposable element and gene expression
Source: PLoS One. 2021 Sep 29;16(9):e0251611. doi: 10.1371/journal.pone.0251611 (PMC8480850; doi:10.1371/journal.pone.0251611)
Supplement: S1 Fig — Gene Set Enrichment Analysis, top 30 activated/suppressed enriched pathways for all conditions. (A) Enriched pathways, both activated and suppressed, determined by GSEA for AD cases. (B) Enriched pathways, both activated and suppressed, determined by GSEA for PSP cases. None of the enriched pathways fall below FDR-corrected p-value < 0.05. (C) Enriched pathways, both activated and suppressed, determined by GSEA for LV- 3R4R with Ab cases. (D) Enriched pathways, both activated and suppressed, determined by GSEA for LV-3R4R with DMSO cases. (E) Enriched pathways, both activated and suppressed, determined by GSEA for LV-4R with Ab cases. (F) Enriched pathways, both activated and suppressed, determined by GSEA for LV-4R with DMSO cases. (G) Enriched pathways, both activated and suppressed, determined by GSEA for LV-3R with Ab cases. (H) Enriched pathways, both activated and suppressed, determined by GSEA for LV-3R with DMSO cases. GSEA was done with settings set to 1000 permutations, a minimum gene set size of 15, a maximum gene set size of 200, and FDR-adjusted p-values. (PDF) [file pone.0251611.s006.pdf]

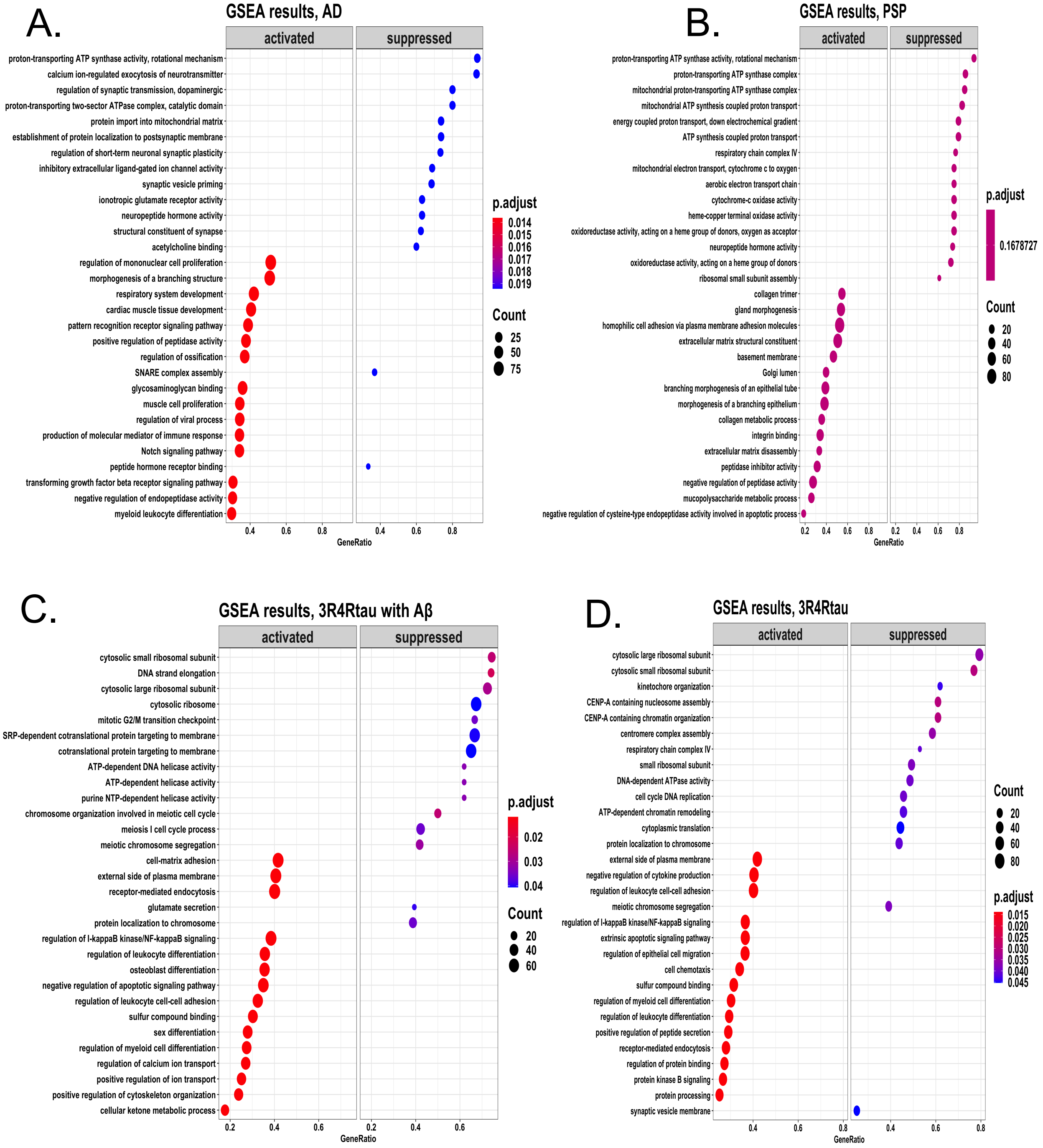
A.
GSEA results, AD
activated
suppressed
proton-transporting ATP synthase activity, rotational mechanism
calcium ion-regulated exocytosis of neurotransmitter
regulation of synaptic transmission, dopaminergic
proton-transporting two-sector ATPase complex, catalytic domain
protein import into mitochondrial matrix
establishment of protein localization to postsynaptic membrane
regulation of short-term neuronal synaptic plasticity
inhibitory extracellular ligand-gated ion channel activity
synaptic vesicle priming
ionotropic glutamate receptor activity
neuropeptide hormone activity
structural constituent of synapse
acetylcholine binding
regulation of mononuclear cell proliferation
morphogenesis of a branching structure
respiratory system development
cardiac muscle tissue development
pattern recognition receptor signaling pathway
positive regulation of peptidase activity
regulation of ossification
SNARE complex assembly
glycosaminoglycan binding
muscle cell proliferation
regulation of viral process
production of molecular mediator of immune response
Notch signaling pathway
peptide hormone receptor binding
transforming growth factor beta receptor signaling pathway
negative regulation of endopeptidase activity
myeloid leukocyte differentiation
0.4
0.6
0.8
0.4
0.6
0.8
GeneRatio
p.adjust
0.014
0.015
0.016
0.017
0.018
0.019
Count
25
50
75
B.
GSEA results, PSP
activated
suppressed
proton-transporting ATP synthase activity, rotational mechanism
proton-transporting ATP synthase complex
mitochondrial proton-transporting ATP synthase complex
mitochondrial ATP synthesis coupled proton transport
energy coupled proton transport, down electrochemical gradient
ATP synthesis coupled proton transport
respiratory chain complex IV
mitochondrial electron transport, cytochrome c to oxygen
aerobic electron transport chain
cytochrome-c oxidase activity
heme-copper terminal oxidase activity
oxidoreductase activity, acting on a heme group of donors, oxygen as acceptor
neuropeptide hormone activity
oxidoreductase activity, acting on a heme group of donors
ribosomal small subunit assembly
collagen trimer
gland morphogenesis
homophilic cell adhesion via plasma membrane adhesion molecules
extracellular matrix structural constituent
basement membrane
Golgi lumen
branching morphogenesis of an epithelial tube
morphogenesis of a branching epithelium
collagen metabolic process
integrin binding
extracellular matrix disassembly
peptidase inhibitor activity
negative regulation of peptidase activity
mucopolysaccharide metabolic process
negative regulation of cysteine-type endopeptidase activity involved in apoptotic process
0.2
0.4
0.6
0.8
0.2
0.4
0.6
0.8
GeneRatio
p.adjust
0.1678727
Count
20
40
60
80
C.
GSEA results, 3R4Rtau with Aβ
activated
suppressed
cytosolic small ribosomal subunit
DNA strand elongation
cytosolic large ribosomal subunit
cytosolic ribosome
mitotic G2/M transition checkpoint
SRP-dependent cotranslational protein targeting to membrane
cotranslational protein targeting to membrane
ATP-dependent DNA helicase activity
ATP-dependent helicase activity
purine NTP-dependent helicase activity
chromosome organization involved in meiotic cell cycle
meiosis I cell cycle process
meiotic chromosome segregation
cell-matrix adhesion
external side of plasma membrane
receptor-mediated endocytosis
glutamate secretion
protein localization to chromosome
regulation of I-kappaB kinase/NF-kappaB signaling
regulation of leukocyte differentiation
osteoblast differentiation
negative regulation of apoptotic signaling pathway
regulation of leukocyte cell-cell adhesion
sulfur compound binding
sex differentiation
regulation of myeloid cell differentiation
regulation of calcium ion transport
positive regulation of ion transport
positive regulation of cytoskeleton organization
cellular ketone metabolic process
0.2
0.4
0.6
0.2
0.4
0.6
GeneRatio
p.adjust
0.02
0.03
0.04
Count
20
40
60
D.
GSEA results, 3R4Rtau
activated
suppressed
cytosolic large ribosomal subunit
cytosolic small ribosomal subunit
kinetochore organization
CENP-A containing nucleosome assembly
CENP-A containing chromatin organization
centromere complex assembly
respiratory chain complex IV
small ribosomal subunit
DNA-dependent ATPase activity
cell cycle DNA replication
ATP-dependent chromatin remodeling
cytoplasmic translation
protein localization to chromosome
external side of plasma membrane
negative regulation of cytokine production
regulation of leukocyte cell-cell adhesion
meiotic chromosome segregation
regulation of I-kappaB kinase/NF-kappaB signaling
extrinsic apoptotic signaling pathway
regulation of epithelial cell migration
cell chemotaxis
sulfur compound binding
regulation of myeloid cell differentiation
regulation of leukocyte differentiation
positive regulation of peptide secretion
receptor-mediated endocytosis
regulation of protein binding
protein kinase B signaling
protein processing
synaptic vesicle membrane
0.4
0.6
0.8
0.4
0.6
0.8
GeneRatio
Count
20
40
60
80
p.adjust
0.015
0.020
0.025
0.030
0.035
0.040
0.045

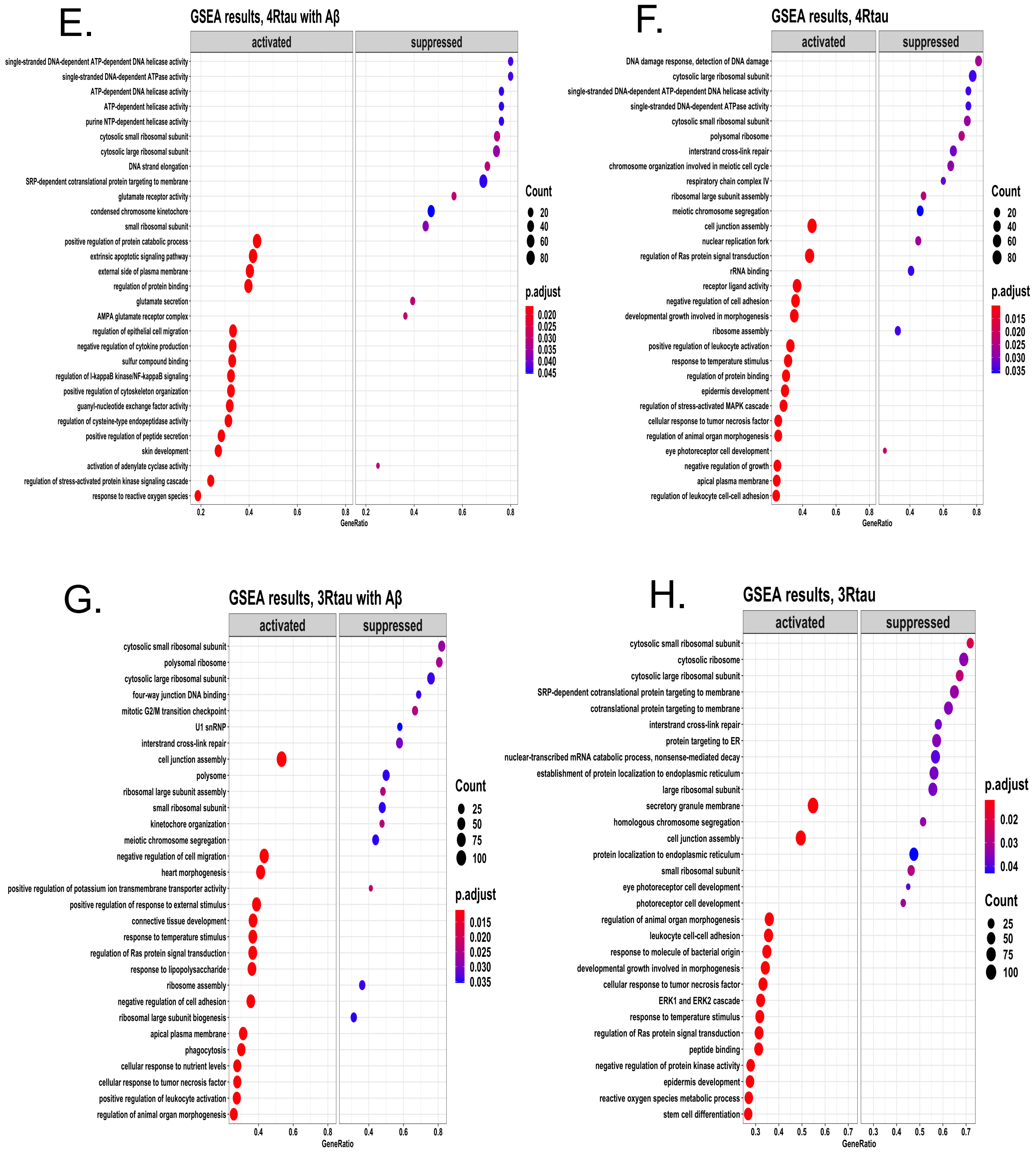

Fig S3. Gene Set Enrichment Analysis, top 30 activated/suppressed enriched pathways for all conditions.

(A) Enriched pathways, both activated and suppressed, determined by GSEA for AD cases. (B) Enriched pathways, both activated and suppressed, determined by GSEA for PSP cases. None of the enriched pathways fall below FDR-corrected p-value < 0.05. (C) Enriched pathways, both activated and suppressed, determined by GSEA for LV-3R4R with Aβ cases. (D) Enriched pathways, both activated and suppressed, determined by GSEA for LV-3R4R with DMSO cases. (E) Enriched pathways, both

activated and suppressed, determined by GSEA for LV-4R with Aβ cases. (F) Enriched pathways, both activated and suppressed, determined by GSEA for LV-4R with DMSO cases. (G) Enriched pathways, both activated and suppressed, determined by GSEA for LV-3R with Aβ cases. (H) Enriched pathways, both activated and suppressed, determined by GSEA for LV-3R with DMSO cases. GSEA was done with settings set to 1000 permutations, a minimum gene set size of 15, a maximum gene set size of 200, and FDR-adjusted p-values.
