# Supplementary material for: Transcriptome analyses reveal tau isoform-driven changes in transposable element and gene expression
Source: PLoS One. 2021 Sep 29;16(9):e0251611. doi: 10.1371/journal.pone.0251611 (PMC8480850; doi:10.1371/journal.pone.0251611)
Supplement: S1 File — (ZIP) [file pone.0251611.s005.zip › S4_Code_and_Quality_Control_Data/QC_data_for_cells.zip!/QC_data_for_cells/cells_Qualimap_multisampleBamQcReport.html]

Qualimap report: Multi-sample BAM QC


Qualimap Report: Multi-sample BAM QC

## Input data and parameters

### Samples (with groups)

|  |  |
| --- | --- |
| Ctrl\_AB1 (Ctrl\_AB) | Ctrl\_AB\_1 |
| 3R\_AB1 (3R\_AB) | 3R\_AB\_1 |
| Ctrl\_1 (Ctrl\_only) | Ctrl\_1 |
| Ctrl\_3 (Ctrl\_only) | Ctrl\_3 |
| 3R4R\_3 (3R4R\_only) | 3R4R\_3 |
| 4R\_AB2 (4R\_AB) | 4R\_AB\_2 |
| 3R\_AB2 (3R\_AB) | 3R\_AB\_2 |
| Ctrl\_AB2 (Ctrl\_AB) | Ctrl\_AB\_2 |
| 3R4R\_AB3 (3R4R\_AB) | 3R4R\_AB\_3 |
| 4R\_1 (4R\_only) | 4R\_1 |
| 4R\_AB1 (4R\_AB) | 4R\_AB\_1 |
| 3R\_AB3 (3R\_AB) | 3R\_AB\_3 |
| 3R4R\_AB2 (3R4R\_AB) | 3R4R\_AB\_2 |
| 3R\_3 (3R\_only) | 3R\_3 |
| 4R\_2 (4R\_only) | 4R\_2 |
| 3R4R\_AB1 (3R4R\_AB) | 3R4R\_AB\_1 |
| 3R\_2 (3R\_only) | 3R\_2 |
| 3R\_1 (3R\_only) | 3R\_1 |
| 3R4R\_1 (3R4R\_only) | 3R4R\_1 |
| Ctrl\_AB3 (Ctrl\_AB) | Ctrl\_AB\_3 |
| 4R\_3 (4R\_only) | 4R\_3 |
| 4R\_AB3 (4R\_AB) | 4R\_AB\_3 |
| 3R4R\_2 (3R4R\_only) | 3R4R\_2 |
| Ctrl\_2 (Ctrl\_only) | Ctrl\_2 |

## Summary

### Globals

|  |  |
| --- | --- |
| Number of samples | 24 |
| Number of groups | 8 |
| Total number of mapped reads | 1,261,537,922 |
| Mean samples coverage | 34.25 |
| Mean samples GC-content | 49.23 |
| Mean samples mapping quality | 248.72 |
| Mean samples insert size | 351.67 |

### Sample statistics

|  |  |  |  |  |  |  |
| --- | --- | --- | --- | --- | --- | --- |
| **Sample** | **Group** | **Coverage mean** | **Coverage std** | **GC percentage** | **Mapping quality mean** | **Insert size median** |
| 3R\_1 | 3R\_only | 43.7092 | 194.3783 | 48.51 | 248.9327 | 350.0 |
| 3R\_2 | 3R\_only | 30.9714 | 145.6464 | 49.11 | 248.5552 | 356.0 |
| 3R\_3 | 3R\_only | 24.7927 | 127.2156 | 48.49 | 248.9956 | 328.0 |
| 3R\_AB1 | 3R\_AB | 31.0161 | 133.3854 | 49.0 | 248.6826 | 310.0 |
| 3R\_AB2 | 3R\_AB | 42.1662 | 193.683 | 49.17 | 248.7933 | 362.0 |
| 3R\_AB3 | 3R\_AB | 34.3796 | 166.4821 | 48.75 | 248.6845 | 369.0 |
| 4R\_1 | 4R\_only | 48.5825 | 222.2154 | 49.95 | 249.3397 | 349.0 |
| 4R\_2 | 4R\_only | 35.6973 | 159.9381 | 48.77 | 248.705 | 333.0 |
| 4R\_3 | 4R\_only | 23.7938 | 108.5679 | 49.14 | 248.7834 | 340.0 |
| 4R\_AB1 | 4R\_AB | 38.147 | 206.7756 | 49.58 | 249.0811 | 383.0 |
| 4R\_AB2 | 4R\_AB | 30.9859 | 131.2901 | 49.11 | 248.5704 | 324.0 |
| 4R\_AB3 | 4R\_AB | 28.6986 | 118.8198 | 50.69 | 248.553 | 330.0 |
| 3R4R\_1 | 3R4R\_only | 42.7849 | 176.9522 | 50.78 | 248.4956 | 365.0 |
| 3R4R\_2 | 3R4R\_only | 33.5543 | 158.2393 | 50.49 | 248.3804 | 347.0 |
| 3R4R\_3 | 3R4R\_only | 39.3655 | 211.7461 | 48.98 | 248.846 | 358.0 |
| 3R4R\_AB1 | 3R4R\_AB | 34.576 | 177.3716 | 49.98 | 248.4646 | 375.0 |
| 3R4R\_AB2 | 3R4R\_AB | 25.5204 | 135.6643 | 48.87 | 248.7657 | 332.0 |
| 3R4R\_AB3 | 3R4R\_AB | 40.935 | 222.1098 | 49.28 | 248.6757 | 388.0 |
| Ctrl\_1 | Ctrl\_only | 36.914 | 169.6275 | 49.09 | 248.6418 | 374.0 |
| Ctrl\_2 | Ctrl\_only | 31.0807 | 170.9311 | 49.33 | 248.5734 | 358.0 |
| Ctrl\_3 | Ctrl\_only | 31.6379 | 182.6717 | 48.07 | 248.7298 | 363.0 |
| Ctrl\_AB1 | Ctrl\_AB | 27.8812 | 115.4417 | 49.39 | 248.7004 | 354.0 |
| Ctrl\_AB2 | Ctrl\_AB | 29.8407 | 136.7925 | 48.84 | 248.6624 | 362.0 |
| Ctrl\_AB3 | Ctrl\_AB | 34.9688 | 165.4367 | 48.26 | 248.786 | 330.0 |

## PCA

## Coverage Across Reference

## Coverage Histogram (0 - 245786X)

## Global Coverage Histogram

## Genome Fraction Coverage

## Duplication Rate Histogram

## Mapped reads GC-content

## Mapped Reads Clipping Profile

## Mapped Reads GC-content Distribution

## Mapping Quality Across Reference

## Mapping Quality Histogram

## Insert Size Across Reference

## Insert Size Histogram

### Contents

- Input data & parameters
- Summary
- PCA
- Coverage Across Reference
- Coverage Histogram (0 - 245786X)
- Global Coverage Histogram
- Genome Fraction Coverage
- Duplication Rate Histogram
- Mapped reads GC-content
- Mapped Reads Clipping Profile
- Mapped Reads GC-content Distribution
- Mapping Quality Across Reference
- Mapping Quality Histogram
- Insert Size Across Reference
- Insert Size Histogram

2020/01/08 12:20:38

Generated by QualiMap v.2.2.2-dev
