# Supplementary material for: Transcriptome analyses reveal tau isoform-driven changes in transposable element and gene expression
Source: PLoS One. 2021 Sep 29;16(9):e0251611. doi: 10.1371/journal.pone.0251611 (PMC8480850; doi:10.1371/journal.pone.0251611)
Supplement: S1 File — (ZIP) [file pone.0251611.s005.zip › S4_Code_and_Quality_Control_Data/QC_data_for_cells.zip!/QC_data_for_cells/cells_Qualimap_RNAseqQC_multiqc_report.html]

MultiQC Report


# Toggle navigation v1.9

Loading report..

- General Stats
- QualiMap
  - Genomic origin of reads
  - Gene Coverage Profile

Toolbox

### MultiQC Toolbox

#### Apply Highlight Samples

+

Regex mode off
help
 Clear

#### Apply Rename Samples

+

Click here for bulk input.

Paste two columns of a tab-delimited table here (eg. from Excel).

First column should be the old name, second column the new name.

Add

Regex mode off
help
 Clear

#### Apply Show / Hide Samples

Hide matching samples

Show only matching samples

+

Regex mode off
help
 Clear

#### Export Plots

- Images
- Data

px

px

Aspect ratio

PNG
JPEG
SVG

Plot scaling

X

Download the raw data used to create the plots in this report below:

Format:

Tab-separated
Comma-separated
JSON

Note that additional data was saved in `multiqc_data` when this report was generated.

---

##### Choose Plots

 All
 None


---


   Download Plot Images

If you use plots from MultiQC in a publication or presentation, please cite:

> **MultiQC: Summarize analysis results for multiple tools and samples in a single report**  
> *Philip Ewels, Måns Magnusson, Sverker Lundin and Max Käller*  
> Bioinformatics (2016)  
> doi: 10.1093/bioinformatics/btw354  
> PMID: 27312411

#### Save Settings

You can save the toolbox settings for this report to the browser.

 Save


---

#### Load Settings

Choose a saved report profile from the dropdown box below:

[ select ]

Load
 Delete
 Set default
 Clear default

#### About MultiQC

This report was generated using MultiQC, version 1.9

You can see a YouTube video describing how to use MultiQC reports here:
https://youtu.be/qPbIlO\_KWN0

For more information about MultiQC, including other videos and
extensive documentation, please visit http://multiqc.info

You can report bugs, suggest improvements and find the source code for MultiQC on GitHub:
https://github.com/ewels/MultiQC

MultiQC is published in Bioinformatics:

> **MultiQC: Summarize analysis results for multiple tools and samples in a single report**  
> *Philip Ewels, Måns Magnusson, Sverker Lundin and Max Käller*  
> Bioinformatics (2016)  
> doi: 10.1093/bioinformatics/btw354  
> PMID: 27312411

A modular tool to aggregate results from bioinformatics analyses across many samples into a single report.

#### JavaScript Disabled

MultiQC reports use JavaScript for plots and toolbox functions. It looks like
you have JavaScript disabled in your web browser. Please note that many of the report
functions will not work as intended.

Loading report..

Report
generated on 2020-11-26, 15:36
based on data in:

- `/Volumes/Betelgeuse/Colossus/Ensembl_QC/Ensembl_rnaseq_qc_results/3R4R_1`
- `/Volumes/Betelgeuse/Colossus/Ensembl_QC/Ensembl_rnaseq_qc_results/3R4R_2`
- `/Volumes/Betelgeuse/Colossus/Ensembl_QC/Ensembl_rnaseq_qc_results/3R4R_3`
- `/Volumes/Betelgeuse/Colossus/Ensembl_QC/Ensembl_rnaseq_qc_results/3R4R_AB_1`
- `/Volumes/Betelgeuse/Colossus/Ensembl_QC/Ensembl_rnaseq_qc_results/3R4R_AB_2`
- `/Volumes/Betelgeuse/Colossus/Ensembl_QC/Ensembl_rnaseq_qc_results/3R4R_AB_3`
- `/Volumes/Betelgeuse/Colossus/Ensembl_QC/Ensembl_rnaseq_qc_results/3R_1`
- `/Volumes/Betelgeuse/Colossus/Ensembl_QC/Ensembl_rnaseq_qc_results/3R_2`
- `/Volumes/Betelgeuse/Colossus/Ensembl_QC/Ensembl_rnaseq_qc_results/3R_3`
- `/Volumes/Betelgeuse/Colossus/Ensembl_QC/Ensembl_rnaseq_qc_results/3R_AB_1`
- `/Volumes/Betelgeuse/Colossus/Ensembl_QC/Ensembl_rnaseq_qc_results/3R_AB_2`
- `/Volumes/Betelgeuse/Colossus/Ensembl_QC/Ensembl_rnaseq_qc_results/3R_AB_3`
- `/Volumes/Betelgeuse/Colossus/Ensembl_QC/Ensembl_rnaseq_qc_results/4R_1`
- `/Volumes/Betelgeuse/Colossus/Ensembl_QC/Ensembl_rnaseq_qc_results/4R_2`
- `/Volumes/Betelgeuse/Colossus/Ensembl_QC/Ensembl_rnaseq_qc_results/4R_3`
- `/Volumes/Betelgeuse/Colossus/Ensembl_QC/Ensembl_rnaseq_qc_results/4R_AB_1`
- `/Volumes/Betelgeuse/Colossus/Ensembl_QC/Ensembl_rnaseq_qc_results/4R_AB_2`
- `/Volumes/Betelgeuse/Colossus/Ensembl_QC/Ensembl_rnaseq_qc_results/4R_AB_3`
- `/Volumes/Betelgeuse/Colossus/Ensembl_QC/Ensembl_rnaseq_qc_results/Ctrl_1`
- `/Volumes/Betelgeuse/Colossus/Ensembl_QC/Ensembl_rnaseq_qc_results/Ctrl_2`
- `/Volumes/Betelgeuse/Colossus/Ensembl_QC/Ensembl_rnaseq_qc_results/Ctrl_3`
- `/Volumes/Betelgeuse/Colossus/Ensembl_QC/Ensembl_rnaseq_qc_results/Ctrl_AB_1`
- `/Volumes/Betelgeuse/Colossus/Ensembl_QC/Ensembl_rnaseq_qc_results/Ctrl_AB_2`
- `/Volumes/Betelgeuse/Colossus/Ensembl_QC/Ensembl_rnaseq_qc_results/Ctrl_AB_3`

---

×
don't show again

**Welcome!** Not sure where to start?  
Watch a tutorial video
  *(6:06)*

## General Statistics

 Copy table

 Configure Columns

 Sort by highlight

 Plot
Showing ^24^/_24_ rows and ^2^/_2_ columns.

| Sample Name | 5'-3' bias | M Aligned |
| --- | --- | --- |
| 3R4R\_1 | 1.16 | 31.4 |
| 3R4R\_2 | 1.14 | 25.6 |
| 3R4R\_3 | 1.16 | 31.3 |
| 3R4R\_AB\_1 | 1.17 | 27.0 |
| 3R4R\_AB\_2 | 1.18 | 20.1 |
| 3R4R\_AB\_3 | 1.17 | 31.8 |
| 3R\_1 | 1.14 | 34.4 |
| 3R\_2 | 1.16 | 23.5 |
| 3R\_3 | 1.17 | 20.5 |
| 3R\_AB\_1 | 1.14 | 24.7 |
| 3R\_AB\_2 | 1.16 | 33.6 |
| 3R\_AB\_3 | 1.19 | 27.0 |
| 4R\_1 | 1.09 | 39.0 |
| 4R\_2 | 1.12 | 27.8 |
| 4R\_3 | 1.14 | 18.2 |
| 4R\_AB\_1 | 1.14 | 29.2 |
| 4R\_AB\_2 | 1.19 | 24.5 |
| 4R\_AB\_3 | 1.15 | 21.4 |
| Ctrl\_1 | 1.16 | 28.1 |
| Ctrl\_2 | 1.18 | 25.5 |
| Ctrl\_3 | 1.18 | 25.4 |
| Ctrl\_AB\_1 | 1.15 | 22.1 |
| Ctrl\_AB\_2 | 1.22 | 24.0 |
| Ctrl\_AB\_3 | 1.18 | 27.7 |

×

#### General Statistics: Columns

Uncheck the tick box to hide columns. Click and drag the handle on the left to change order.

Show All
Show None

| Sort | Visible | Group | Column | Description | ID | Scale |
| --- | --- | --- | --- | --- | --- | --- |
| \|\| |  | QualiMap | 5'-3' bias | 5'-3' bias | `5_3_bias` | None |
| \|\| |  | QualiMap | M Aligned | Reads Aligned (millions) | `reads_aligned` | read\_count |

Close

## QualiMap

QualiMap is a platform-independent application to facilitate the quality control of alignment sequencing data and its derivatives like feature counts.

### Genomic origin of reads Help

Classification of mapped reads as originating in exonic, intronic or intergenic regions. These can be displayed as either the number or percentage of mapped reads.

There are currently three main approaches to map reads to transcripts in an
RNA-seq experiment: mapping reads to a reference genome to identify expressed
transcripts that are annotated (and discover those that are unknown), mapping
reads to a reference transcriptome, and *de novo* assembly of transcript
sequences (Conesa et al. 2016).

For RNA-seq QC analysis, QualiMap can be used to assess alignments produced by
the first of these approaches. For input, it requires a GTF annotation file
along with a reference genome, which can be used to reconstruct the exon
structure of known transcripts. This allows mapped reads to be grouped by
whether they originate in an exonic region (for QualiMap, this may include
5′ and 3′ UTR regions as well as protein-coding exons), an intron,
or an intergenic region (see the Qualimap 2 documentation).

The inferred genomic origins of RNA-seq reads are presented here as a bar graph
showing either the number or percentage of mapped reads in each read dataset
that have been assigned to each type of genomic region. This graph can be used
to assess the proportion of useful reads in an RNA-seq experiment. That
proportion can be reduced by the presence of intron sequences, especially if
depletion of ribosomal RNA was used during sample preparation (Sims et al. 2014). It can also be reduced by off-target
transcripts, which are detected in greater numbers at the sequencing depths
needed to detect poorly-expressed transcripts (Tarazona et al. 2011).

Counts
Percentages

loading..

---

### Gene Coverage Profile Help

Mean distribution of coverage depth across the length of all mapped transcripts.

There are currently three main approaches to map reads to transcripts in an
RNA-seq experiment: mapping reads to a reference genome to identify expressed
transcripts that are annotated (and discover those that are unknown), mapping
reads to a reference transcriptome, and *de novo* assembly of transcript
sequences (Conesa et al. 2016).

For RNA-seq QC analysis, QualiMap can be used to assess alignments produced by
the first of these approaches. For input, it requires a GTF annotation file
along with a reference genome, which can be used to reconstruct the exon
structure of known transcripts. QualiMap uses this information to calculate the
depth of coverage along the length of each annotated transcript. For a set of
reads mapped to a transcript, the depth of coverage at a given base position is
the number of high-quality reads that map to the transcript at that position
(Sims et al. 2014).

QualiMap calculates coverage depth at every base position of each annotated
transcript. To enable meaningful comparison between transcripts, base positions
are rescaled to relative positions expressed as percentage distance along each
transcript (*0%, 1%, …, 99%*). For the set of transcripts with at least
one mapped read, QualiMap plots the cumulative mapped-read depth (y-axis) at
each relative transcript position (x-axis). This plot shows the gene coverage
profile across all mapped transcripts for each read dataset. It provides a
visual way to assess positional biases, such as an accumulation of mapped reads
at the 3′ end of transcripts, which may indicate poor RNA quality in the
original sample (Conesa et al. 2016).

loading..

**MultiQC v1.9** 
- Written by Phil Ewels,
available on GitHub.

This report uses HighCharts,
jQuery,
jQuery UI,
Bootstrap,
FileSaver.js and
clipboard.js.

×

### Plot Table Data

Select Column

Select Column

Please select two table columns.

Close

×

### Regex Help

Toolbox search strings can behave as regular expressions (regexes). Click a button below to see an example of it in action. Try modifying them yourself in the text box.

`^` (start of string)
`$` (end of string)
`[]` (character choice)
`\d` (shorthand for `[0-9]`)
`\w` (shorthand for `[0-9a-zA-Z_]`)
`.` (any character)
`\.` (literal full stop)
`()` `|` (group / separator)
`*` (prev char 0 or more)
`+` (prev char 1 or more)
`?` (prev char 0 or 1)
`{}` (char num times)
`{,}` (count range)

```
samp_1
samp_1_edited
samp_2
samp_2_edited
samp_3
samp_3_edited
prepended_samp_1
tmp_samp_1_edited
tmpp_samp_1_edited
tmppp_samp_1_edited
#samp_1_edited.tmp
samp_11
samp_11111
```

See regex101.com for a more heavy duty testing suite.

Close
